# Supplementary material for: Chromothripsis during telomere crisis is independent of NHEJ, and consistent with a replicative origin
Source: Genome Res. 2019 May;29(5):737–49. doi: 10.1101/gr.240705.118 (PMC6499312; doi:10.1101/gr.240705.118)
Supplement: Supplemental Material [file supp_gr.240705.118_Supplemental_file_1.zip › contigs/annotated_contigs/DB51/contig.2.DB51_length_636_mean_cov_4.48113207547.docx]

**DB51_length_636_mean_cov_4.48113207547**

```
CCAGAACTTACGGAAGTTGCTTAAATCCTTCAGGTCCTTTGTTGCCTTATGGATAATAATAGCACCTTCATAGCATTGACGTGAAAAGG
 >chr6:164444707-164445036 - E=3e-183
AATAAAATAATGCAAATGAAGCCCACAGTACCAGGCATGTAAGTGCTCAATAAATGTTTGCTACTACTTTTCTGTCCAGATTTTTCAGC

TGAGGATTTGATATTTTTTAAGAACCAGTACTGGAATGACAACTGCCAGGCTTCCGTCAGCTGCCACTGTTCATAGTAAGCACACACAG

GGTTCTCCTCTGCTACTGCACCGATTAAAAATAGATTTTAATTCCCCACAGGACAAAAC|GAA|GTCTCCAATCATATATGATGTTGAC
                                                            >chr6:154450353-154450663 -
ATTGAGAGGAGAGAAAGACCCTCTCACCTTGTTTTATATTGTTTCATATTCAGTAAAAACAACAAGGAAGTAAAACCAAATACAGGCAG
 E=9e-169
CTCAGCGCCAGGCCCGAAACCAGGCCTGGGCCCGCCTGGCCTAAACCCAGTAGTTAAAAATCAACTTATGATTTAGAAGCCGATGTTAT

TCATAGATTCCTTACATTGTATAGAAGAACATTGTTCTGTTCTCTATTGTATAGAAGAACTCCCTGCCCTGTTCTGTTCCTCCCAGACC

ACCGGTGCATGCAGC
```
